# Bioimpacts of dialyzer variety on phosphorus level in Iranian hemodialysis patients

**DOI:** 10.15171/jrip.2016.20

**Published:** 2016-05-30

**Authors:** Aiyoub Pezeshgi, Bahareh Moharrami, Goodarz Kolifarhood, Alireza Sadeghi, Masoud Asadi-Khiavi

**Affiliations:** ^1^Zanjan Metabolic Disease Research Center, Zanjan University of Medical Sciences, Zanjan, Iran; ^2^Department of Internal Medicine, School of Medicine, Zanjan University of Medical Sciences, Zanjan, Iran; ^3^Department of Epidemiology, School of Medicine, Zanjan University of Medical Sciences, Zanjan, Iran; ^4^Zanjan Applied Pharmacology Research Center, Zanjan University of Medical Sciences, Zanjan, Iran; ^5^Department of Pharmacotherapy, School of Pharmacy, Zanjan University of Medical Sciences, Zanjan, Iran

**Keywords:** Hemodialysis, Phosphorus, Dialyzer

## Abstract

**Introduction:** Cardiovascular events are the major cause of death in patients with chronic renal failure. About half of dialysis patients because of reduced phosphorus clearance have hyperphosphatemia. Hyperphosphatemia and following secondary hyperparathyroidism lead to some cardiovascular changes. Hemodialysis (HD) partly removes phosphorus during each dialysis session.

**Objectives:** Presented study was designed to evaluate dialyzer variation effect on phosphorus level as a prognostic factor after dialysis using.

**Materials and Methods:** Six kinds of dialyzer were used for dialysis; low flux (LF) dialyzer (F7 and F8), high flux (HF) dialyzer (F70 and F80) and finally hollow-fiber dialyzers including polyethersulfone (PES) 130 HF and polysulfone (PS) 13 LF. Fifty-seven patients were divided into 6 matched groups included three groups of 10 people and 3 groups of 9 persons in groups: A (F70), B (F80), C (F7), D (F8), E (PES 130 HF) and F (PS 13 LF). Patients were treated for one month with these dialyzers. At the end of the month, blood samples were taken again for phosphorus level before dialysis handling.

**Results:** The mean pre-dialysis serum phosphorus was 5.03, 5.4, 5.2, 4.6, 4.95 and 5.1 mg/dl and the mean phosphorus was 5.43, 5.01, 4.9, 4.18, 4.17 and 5.3 mg/dl after one month of dialysis, respectively in groups A to F without any statistically differences between pre- and after one month dialysis values respectively.

**Discussion:** The findings indicate dialyzer type in the control of serum phosphorus has not been effective in the short-term HD. We suggest a study with more duration time.

**Implication for health policy/practice/research/medical education:**

Dialyzer variety ineffectiveness on serum phosphorus level during short-term survey was revealed in presented study. On the other hand, it is concluded that the routine handling for phosphorus level without any care about dialyzer variety and following sticky situations (e.g. electrolytes and diet disturbances, drug interactions and caring about supplementary doses of needed drugs and so on) is enough for patient treatment at least in short time periods.

## Introduction

Cardiovascular events are the major cause of death in patients with chronic renal failure which 10 to 30 times more inpatients with end-stage renal disease (ESRD) than others. Attention on risk factors of cardiovascular events is essential to reduce them. Despite traditional risk factors (diabetes, hypertension, hyperlipidemia, smoking, aging and male gender), non-traditional risk factors such as proteinuria, hypervolemia, anemia, vitamin D_3_ deficiency, hyper-homocysteinemia, malnutrition, thrombogenic factors, sleep disorders, hyperuricemia, chronic inflammation and hyperphosphatemia increase the risk of cardiovascular events and recently mentioned hyperphosphatemia, is a trouble in hemodialysis (HD) patients with own constitutional risk (1,2). Hyperphosphatemia and following secondary hyperparathyroidism and activated vitamin D_3_ reduction lead to myocardial hypertrophy, coronary artery calcification, increased pulse pressure, increased cardiac after load and cardiomyocytes as well as following fibrosis (3). Pre-dialysis serum phosphorus less than 5.5 mg/dl was associated with the lowest rate of death but levels more than 9 mg/dl and lower than 3 mg/dl were associated with increased mortality. According to the Kidney Disease Outcomes Quality Initiative (KDOQI) recommended in patients with ESRD, serum phosphorus should be maintained between 3.5 mg/dl to 5.5 mg/dl. Additionally obtained by multiplying the phosphorus and calcium must be kept lower than 5.5 mg/dl. Phosphate is an intracellular anion and its transfer rate into the extracellular space is limited. Phosphorus balance is maintained via its renal excretion. Retention of phosphorus in dialysis patients is common and current strategies for overcome on it include dietary phosphorus limitation, intestinal absorption reduction using phosphorus binders (such as calcium carbonate, lanthanum carbonate and sevelamer as well as calcium acetate, magnesium carbonate and aluminum hydroxide binders) and phosphorus extraction by HD. HD removes about 800 mg of phosphorus during each dialysis session in according to pre-dialysis level of it. Prolongation of dialysis session time increases phosphorus uptake as well (4-7). The major problem is that the level of phosphate clearance immediately falls during dialysis because of its presence as intra-cellular ion. Dialyzer efficiency to remove urea is measured with mass transfer area coefficient (KOA). Dialyzer potency of extraction decreases through the enlargement of the molecules (3). Conflicting results on the role of different dialyzers to control the amount of phosphorus in HD patients have reported in some studies. Some studies on HD efficiency such as increasing in number of dialysis sessions, prolonging in each HD session and increasing in HD flow rate has been experienced already. Beside of phosphorus control by dietary phosphorus restriction, phosphate binders and in according to dialysis sessions increase, the high flux dialyzers are commonly used without consequence hyperphosphatemia liberate (5).

## Objectives

Presented study was designed to evaluate dialyzer variation effect on phosphorus level as a prognostic factor after dialysis using.

## Materials and Methods

### Dialyzers

This study was performed as a double-blind quasi-experimental study. Six kinds of dialyzer were used for dialysis; low flux (LF) dialyzer (F7 and F8), high flux (HF) dialyzer (F70 and F80) and finally hollow-fiber dialyzers including polyethersulfone (PES) 130 HF and polysulfone (PS) 13 LF.

### Patients

Inclusion criteria for enrolling to the study were; appropriate conditions for dialysis performance (e.g. arteriovenous fistula, permanent graft or catheter for dialysis), three dialysis sessions per week, 4 hours for each session of dialysis, dialysis who had a history of at least one year and finally, obtaining written informed consent. Patients with acute renal failure, dialysis patients that their blood pressure was reduced by more than two times during the first month, change the location of the anatomical access for dialysis and cases with phosphorus level higher than 7 mg/dl were excluded from the study.

### Study design

Finally, 57 cases were enrolled in this study. Patients were divided into 6 groups included 3 groups of 10 people and 3 groups of 9 persons in groups: A (F70), B (F80), C (F7), D (F8), E (PES 130 HF) and F (PS 13 LF) by matched age, sex, serum phosphorus level and weight at the initiation of study. At baseline, the patients through an artery, before the start of dialysis were sampled. Serum phosphorus, calcium, creatinine and urea were tested by one operator as well as a unique machine for all experiments. Weight and nutritional condition were obtained for each case as well. Patients were treated for one month with any dialyzer. At the end of the month, blood samples were taken again for phosphorus level before dialysis performance.

### Ethical issues

The research followed the tenets of the Declaration of Helsinki; detailed design of the study was explained to the patients and their families and/or legal guardians. The written informed consent was obtained from all subjects and their legal executors. Ethical permission was obtained, all information remained confidential and mural aspects of study were accepted in details of the plan adopted in the Medical Ethics Committee of Zanjan University of Medical Sciences.

### Statistical analysis

Kolmogorov–Smirnov test was used to confirm the normal distribution of the samples. *T* tests and one-way analysis of variance (ANOVA) were performed and P value <0.05 was considered significant. During the study, each patient with a dialyzer with a constant flow of solution was placed on HD. The weight of the patient to determine the ultrafiltration was determined at the start of each meeting. There was not alteration in calcium level, Rocaltrol measure, and diet properties as well as dialysis period prolongation during study period.

## Results

### Enrolled cases

Fifty-seven cases of HD patients had inclusion criteria (24 female and 33 male). Nine patients were excluded (three cases expired, renal transplants were done for one case and phosphorus serum level of five cases was above 7 mg/dl).

### Phosphorus level values

The values of pre-dialysis phosphorus level and corresponding values of phosphorus at the day of thirtieth after dialysis in groups A to F were shown in Table 1. Findings did not show statistically differences between pre- and post-dialysis phosphorus level in any type of dialyzers (*P*>0.5).

**Table 1:** Dialyzers impact on serum phosphorus in HD patients at the end of one month

**Groups**	**Number of cases**	**Types of dialyzers**	**df**	**The mean values of phosphorus serum level (mg/dl)**		***P *** ***value*** ^a^
				**At the first day of HD**	**At the 30th day of HD**	
A	8	F70	7	5.03	5.43	0.53
B	9	F80	8	5.4	5.1	0.47
C	8	F7	7	5.2	4.9	0.71
D	9	F8	8	4.6	4.18	0.22
E	9	PES130HF	8	4.95	4.17	0.17
F	10	PS13LF	9	5.1	5.3	0.57

^a^*P* value lower than 0.05 was considered as statistically significant.

## Discussion

Hyperphosphatemia handling is a major clinical challenge in dialysis patients. Phosphate levels are higher than normal in nearly 50% of HD patients despite nutritional limitations and phosphate binders using (6,7). Some studies showed increasing in the pump rotation of HD machine had a beneficial effect on serum phosphorus extraction (8). In one study it was shown that daily HD provided reducing in serum phosphorus levels, left ventricular hypertrophy reduction, resulting in better control of extracellular fluid volume, blood pressure and finally improvement of anemia in patients (9). HD filtration impacts on serum phosphorus removal shown in a large number of studies and even though using of phosphate binders were limited and in several instances, more phosphate was added to their dietary in the cases of necessities. Effectiveness of daily HD in removing phosphorus has been proven too (10-12).

## Conclusion

The findings indicate filter type in the control of serum phosphorus has not been effective in the short-term HD. Therefore, due to some similar studies, we suggest a study with more duration time to assess the impact of the dialyzer on phosphorus levels.

## Limitations of study

The limitations of study including lack of timely referral of patients for plasma phosphorus testing as well as cases address and telephone number alteration and finally incomplete or improper consumption of drugs by patients were considered at the beginning of trial as well.

## Authors’ contribution

AP; scientific writing, principal co-author, coordinator. BM; second co-author, help in scientific writing and patients detecting. GK; statistical analysis. AS; second coordinator. MAK; principal author, corresponding author.

## Funding/Support

This article is extracted from residential thesis of Bahareh Moharrami. This study was supported by a grant from Zanjan University of Medical Sciences (No. a-12-627-2, 2014).

## Conflicts of interest

The authors declare no conflict of interest.

## Ethical considerations

Ethical issues (including plagiarism, misconduct, data fabrication, falsification, double publication or submission, redundancy) have been completely observed by the authors.
